# Declining well-being during the COVID-19 pandemic reveals US social inequities

**DOI:** 10.1371/journal.pone.0254114

**Published:** 2021-07-08

**Authors:** Krishna C. Bathina, Marijn ten Thij, Danny Valdez, Lauren A. Rutter, Johan Bollen

**Affiliations:** 1 Luddy School of Informatics, Computing, and Engineering, Indiana University, Bloomington, IN, United States of America; 2 Delft Institute of Applied Mathematics, Delft University of Technology, Delft, The Netherlands; 3 School of Public Health, Indiana University, Bloomington, IN, United States of America; 4 Psychological and Brain Sciences, Indiana University, Bloomington, IN, United States of America; University of São Paulo, BRAZIL

## Abstract

**Background:**

The COVID-19 pandemic led to mental health fallout in the US; yet research about mental health and COVID-19 primarily rely on samples that may overlook variance in regional mental health. Indeed, between-city comparisons of mental health decline in the US may provide further insight into how the pandemic is disproportionately affecting at-risk groups.

**Purpose:**

This study leverages social media and COVID-19-city infection data to measure the longitudinal (January 22- July 31, 2020) mental health effects of the COVID-19 pandemic in 20 metropolitan areas.

**Methods:**

We used longitudinal VADER sentiment analysis of Twitter timelines (January-July 2020) for cohorts in 20 metropolitan areas to examine mood changes over time. We then conducted simple and multivariate Ordinary Least Squares (OLS) regressions to examine the relationship between COVID-19 infection city data, population, population density, and city demographics on sentiment across those 20 cities.

**Results:**

Longitudinal sentiment tracking showed mood declines over time. The univariate OLS regression highlighted a negative linear relationship between COVID-19 city data and online sentiment (β = -.017). Residing in predominantly white cities had a protective effect against COVID-19 driven negative mood (β = .0629, p < .001).

**Discussion:**

Our results reveal that metropolitan areas with larger communities of color experienced a greater subjective well-being decline than predominantly white cities, which we attribute to clinical and socioeconomic correlates that place communities of color at greater risk of COVID-19.

**Conclusion:**

The COVID-19 pandemic is a driver of declining US mood in 20 metropolitan cities. Other factors, including social unrest and local demographics, may compound and exacerbate mental health outlook in racially diverse cities.

## Introduction

Mental health reports indicate that the US population experienced increased psychological distress and worsening of preexisting psychiatric conditions as a result of the COVID-19 pandemic [1, 2]. The Centers for Disease Control and Prevention (CDC) conducted a national survey wherein 40% of US adults reported experiencing increases in anxiety/depression (31%), trauma/stressor-related disorders (26%), substance abuse (13%), suicidal ideation (11%), or a combination of these issues [3]). Global studies indicate that these deleterious mental health effects may worsen over time [4, 5]. An early longitudinal study of the first six weeks of lockdown in the UK showed worrisome increases in suicidal ideation, especially in young adults [4]. Similarly, in the US, 53% adults in mid-July (up from 32% in mid-March) reported difficulty performing daily activities such as sleeping (36%) and eating (32%), along with increases in alcohol and substance use (12%) [6]. Social media-based studies about COVID-19-related mental health note similar trends, including decline in subjective well-being over time [7, 8].

While these studies provide strong indications of possible population-wide declines of mental health associated with the COVID-19 pandemic, there are gaps to address. First, many observations are conducted at either a national level [3], or among very specific groups, such as front-line healthcare workers [9]. While national and group-specific comparisons are vital dimensions of pandemic-related mental health, they may overlook an important source of variance in the relationship: metropolitan composition [10]. Indeed, densely populated cities are prone to greater viral spread [11]. Furthermore, high-density urban environments, particularly in the US, also contain more diverse populations that are prone to more severe COVID-19 infection, such as the Black and Latinx communities, who are also subject to income inequity [12] and healthcare disparities [13]. However, metropolitan areas are not homogeneous. Some are far less densely populated and characterized by proportionally small representation of racial and ethnic minorities.

Between-city comparisons are crucial to document the effects of urban disparities on the relationship between mental health and COVID-19 in US urban environments. However, making multi-city comparisons through surveys can be costly and inefficient [14]. Longitudinal survey tracking is also prone to measurement error and non-generalizable samples [15] that limit the generalizability of conclusions [16, 17]. By contrast, social media offers a large-scale, real-time record (in text and images) of the activities, thoughts, and preoccupations of more than 70% of the US population [18]. As such, it offers unique opportunities to track population-level mood and sentiment over time and space (focusing on specific geographic locations).

Recent research indicates that high quality, spatially aggregated estimates of subjective well-being (SWB), a widely used proxy for mental health, can be successfully and meaningfully extracted from social media data [19]. Social media also remains the most efficient means of procuring real-time data to map in-the-moment mood and sentiment, and consequently an important tool to study longitudinal changes of these crucial societal indicators over time. Consequently, social media data has been leveraged extensively to study a range of psychosocial phenomena, including sleep [20], mental health [21, 22], and resilience amid natural disaster by geographic region [23, 24]. Social media data is further strengthened when combined with traditional quantitative modeling. Stated differently, social media data extends beyond words. Much of the metadata that is derived from social media data is in the form of, or can be translated to, quantitative and numerical data, and can be included in regression or other General Linear Model (GLM). These metadata can be further leveraged with other variables about persons, communities, or populations to draw nuanced conclusions about real-time issues. Therefore, leveraging geocoded features of social media data, for example, with quantitative modeling could provide robust insights about mood and SWB across urban environments during the COVID-19 pandemic.

Herein, we leverage social media data and US COVID-19-related city data to study the association between the COVID-19 pandemic and decreased mental health by modeling the relationship between SWB (a well-known proxy for mental health [19, 25]), city-level infection rates in 20 US metropolitan areas, their population number, population density, and demographic composition. In prior studies, SWB is known to fluctuate in response to natural experiments. Although some survey-based studies show SWB can improve after significant events overtime [26, 27], we hypothesize that SWB among the US population will decrease over time given the compounding nature of a global pandemic and social unrest that occurred in 2020. Specifically, in this study, we measure the mental health effects of the COVID-19 pandemic, respecting methodological challenges associated with the use of this data, and compare outcomes across these cities, accounting for the effects of city characteristics on the strength of relationships derived from OLS regressions and multivariate regression.

## Methods

### User timeline data

We collected a large set of Tweet identifiers (Tweet IDs) for all COVID-19-relevant tweets that originated in the US posted after January 22, 2020 from an open-access repository (see Chen and colleagues [28] for more information about repository data collection methods). We obtained data corresponding to each Tweet ID from Twitter’s Application Programming Interface (API), including the tweet’s text content, associated metadata including the tweet’s corresponding user ID, and geographic information about these same users. Using the geographic data, we identified cohorts of active Twitter users in the 20 US metropolitan areas that at the time of writing were particularly affected by COVID-19 [29], namely San Francisco, Nashville, Seattle, Indianapolis, Boston, Denver, Washington DC, Charlotte, Cleveland, Las Vegas, Atlanta, Baltimore, New Orleans, Houston, Philadelphia, New York, Los Angeles, Chicago, Miami, and Detroit. We removed accounts that were deemed to originate from bots or institutions from this data, retaining 289,211 Twitter users. We then downloaded the individual timelines of each of those users (3,200 most recent tweets, time-sorted by date posted by individual) for an analytical sample of n = 109,409,989 tweets. We subjected the tweets in these individual geolocated timelines to a sentiment analysis to record changes in sentiment for the entire cohort in each specific city between January 22 through July 31, 2020. Hereafter, we refer to this dataset as the user-timeline data. Our data collection and analysis efforts adhered strictly to Twitter’s privacy and data use agreements.

#### VADER sentiment analysis

Sentiment analysis refers to a series of supervised or unsupervised Machine Learning (ML) and Natural Language Processing (NLP) techniques that extract affective polarity or emotional indicators from text. For this study, we used the open-source Valence Aware Dictionary and sEntiment [*sic*] Reasoner (VADER) sentiment tool [30] which was shown to provide the highest prediction accuracy in an extensive survey of sentiment analysis tools for Twitter data [31]. Simply, the VADER algorithm reviews text (i.e., user timelines) and rates its overall polarity (i.e, positivity and negativity) based on the words used, their order, a variety of grammatical features indicating negation, hedging, and magnification, punctuation, and other relevant features commonly found in online language. VADER scores can range from -.99, denoting high negative polarity; and +.99 denoting high positive polarity. VADER scores can also be 0, which denotes neutral polarity. We applied VADER to the user-timeline data to draw conclusions about changes in SWB for each city cohort over the specified period, as a basis for inferences about mental health (i.e., low VADER scores equate to lower levels of subjective well-being). Regarding SWB inferences drawn from VADER scores, high negative VADER scores (e.g., -.99) denote lower SWB; high positive VADER scores (e.g., +.99) denote better SWB.

#### COVIDCast data

COVIDCast is a data repository and API that contains COVID-19 related data in the US, including hospital visits, deaths, number of cases per 1,000 people, among others [32]. For our study, we downloaded the number of new cases per 1,000 individuals (January 22 through July 31, 2020) in the 20 US cities most affected by COVID-19. We used this data to determine if sentiment is predicted by the rolling average of COVID-19 cases within these 20 cities. We also extracted population, population density, and demographic makeup of each city for further analysis, including how those variables may also affect user sentiment over time. Hereafter, we refer to this data as the COVIDCast Data. Our use of COVIDCast data adhered to the APIs terms of service.

#### OLS regression with jackknife resampling

We ran linear and multivariate ordinary least squares regressions to predict sentiment by the total number of COVID-19 cases in 20 metropolitan areas. For our dependent variable (SWB as measured by average VADER sentiment, which was treated as a continuous variable) we divided our user-timeline data into their respective city\ metropolitan area of residence and calculated the cohort’s average VADER sentiment from January 22 through July 31, 2020. The independent variables used in our regression model included: (1) metropolitan population, (2) population density, (3) city demographics (a dichotomized White/non-White percentage variable), and (4) the average number of COVID-19 cases per 1,000 in each city, which we retrieved from the COVIDCast API. Additionally, to calculate and estimate a confidence interval (CI) about the ***R***^***2***^ coefficient of determination, we applied a jackknife resampling approach to calculate a new ***R***^***2***^ 20 times at ***(N***_***cities***_***− 1)*** (i.e. removing a different city with each iteration (e.g 20 cities minus Houston; 20 cities minus Seattle, etc.) We then calculated the 2.5th and 97.5th percentile for the distribution of ***R***^***2***^ values.

We note that all data collection and analyses undertaken in this study complied with the user guidelines for ethical data use. Additionally, because our study involved no human interaction, and involved secondary data analysis, this study was exempt from review by the Institutional Review Board (IRB). This includes all avenues by which we procured data, including Twitter and the COVIDCast API.

## Results

### COVID-19 cases and SWB

We regressed SWB levels on the log10 of the average number of COVID-19 cases per 1,000 people in 20 metropolitan areas and observed a significant negative relationship between SWB levels and COVID-19 cases ***(β = -*.*017*, *R***^***2***^ ***=* .*3769*, *p <* .*005***). A jackknife resampling approach indicated that the 95% CI of the ***R***^***2***^ is [.282, .443]. The model suggests approximately 38% of the total variance in average VADER scores can be explained by COVID-19 prevalence alone. The linear fit is shown below. For a full regression table, please see S1 Table.


$$SENTIMENT=0.106-0.017*{LOG}_{10}(CASES)$$

### Population density, demographics, and population

**Fig 1:**
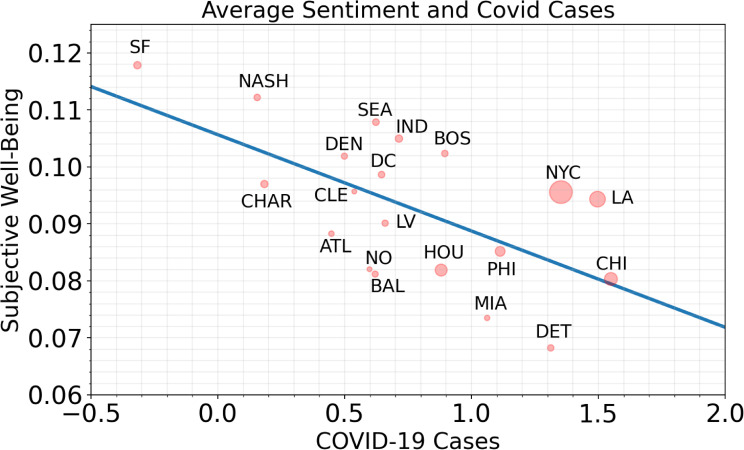
Average VADER score vs observed COVID-19 cases. The blue line displays the line of best fit for all data points. The size of the node is relative to the population size of the cities shown. The negative trajectory suggests that VADER scores are inversely correlated with the mean 7-day average of new COVID-19 cases per 1,000 people.

We then performed a multivariate regression with three additional independent variables on SWB: (1) population density, (2) dichotomized city demographics (i.e. white/non-white), and (3) metropolitan population. We again observed a statistically significant negative relationship between sentiment and log_10_ COVID-19 cases (***β = -*.*0122*, *p =* .*006***). City demographics was also statistically significant (***β*** = .***0629*, *p <* .*001***). Population and population density were not statistically significant. Despite small beta-weights per significant IV, our combined model explained 85% of variance ***(R***^***2***^ ***=* .*847*, *F(4*, *20) = 20*.*73*, *p <* .*001)*.** A Durbin-Watson test for auto-correlation indicated a value of 2.208, hence we conclude there is no auto-correlation between residuals in our model. The linear fit is shown below. For a full regression table, please see S2 Table.


$$SENTIMENT=.073-.012*{LOG}_{10}(CASES)+.063*WHITE\;POPULATION$$

(Fig 1). plots the 20 cities with the X-axis representing the mean value of the 7-day average signal of new COVID-19 cases, and the Y-axis representing average VADER score. The line of best fit is drawn to illustrate the inverse relationship between SWB levels and COVID-19 cases, and indicate between-city differences in SWB relative to COVID-19 cases. For example, San Francisco had high observed SWB and lower COVID-19 cases; Detroit had lower SWB and higher confirmed COVID-19 cases.

### Longitudinal SWB tracking (January-July 2020)

**Fig 2:**
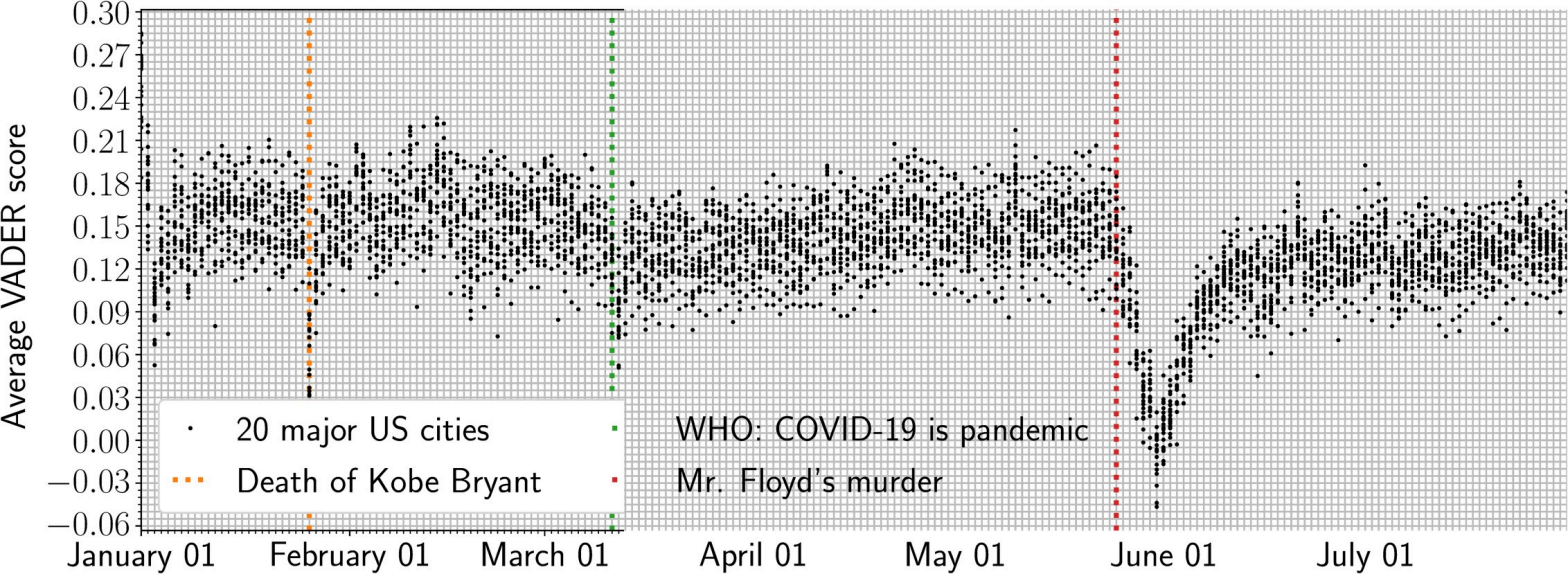
Average longitudinal VADER scores across 20 US cities. Dotted lines represent notable declines in sentiment spurred by 3 acute events: (1) Kobe Bryant’s death, (2) when the World Health Organization (WHO) declared COVID-19 a pandemic, and (3) Mr. George Floyd’s murder. The distribution of sentiment values indicates a sharper drop of SWB with each subsequent event, including a longer time to stabilize at slightly lower levels than before, possible indicating increasing hysteresis effect on societal mood. The effect is particularly pronounced after Mr. Floyd’s murder.

We applied the VADER tool to the user-timeline data and calculated daily average sentiment scores between January 22, through July 31, 2020. In this period, we saw three natural experiments [33] occur in the form of three events: (1) Kobe Bryant’s death (January 26, 2020); (2) when the WHO declared COVID-19 a pandemic (March 11, 2020); and (3) the murder of George Floyd, an African American man, at the hands of a white police officer (May 25, 2020). (Fig 2) plots daily average sentiment time-series for all cities.

**Fig 3:**
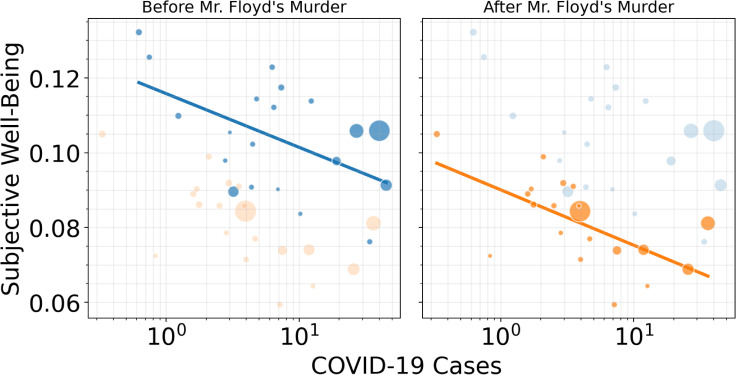
**Average VADER score relative to COVID-19 cases highlighted before (blue) and after Mr. Floyd’s murder (orange) with OLS linear fit.** The size of each bubble indicates the relative city size (population). Although overall SWB decreased after Mr. Floyd’s murder, as indicated by a downward shift in the regression line, the linear relationship between COVID-19 and SWB remains constant.

After observing the precipitous decline in sentiment following Mr. Floyd’s murder and subsequent social unrest [34] we tested if the mentioned events had an effect on the linear relationship between COVID-19 and SWB. (Fig 3) shows a scatterplot of COVID-19 and SWB before and after Mr. Floyd’s murder, with linear fits to gauge the relationship between COVID-19 cases and SWB.

## Discussion

Declines in SWB are consistently negatively tied to confirmed COVID-19 cases across 20 US metropolitan areas. A longitudinal analysis of SWB indicates that the effects of COVID-19 on population mental health are time-variant and compounded by several characteristics including demographic makeup and acute non-COVID-19 related events. Below, we discuss our findings within a public health and medical context, noting important implications for policy and future research.

### COVID-19 and decreased SWB

Across 20 US metropolitan areas, we found an inverse relationship between COVID-19 infection and SWB. The small beta weight in our base regression model, coupled with a high ***R***^***2***^ coefficient of determination, may suggest the pandemic’s effect on SWB is not sudden, but incurred incrementally over time. Gradual SWB declines are likely due to several secondary psychosocial effects associated with higher (and growing) infection rates that compound and exacerbate poor mood over an extended period. Stated differently, a small beta-weight may indicate a marginal effect. Yet, if the effect continues over an extended period, then we enter a period of exasperation. This incremental, long-term effect is supported by a robust effect size (84% in the multivariate model) which suggests changes in mood can be mostly explained by COVID-19 cases and demographic makeup. Incremental devaluation in mood can be seen in documented behaviors throughout 2020, regardless of area of residence. For example, between January and July 2020, state-imposed quarantine measures led to communal feelings of isolation and disconnectedness [35]. Continuously growing infection rates across the US also may have led to collective anxiety about the virus (3), which may have been worsened by increased news consumption [36] and constant media coverage, with varying accuracy [37]. Additionally, as unemployment ballooned to a record 14.7% in April 2020, it is likely economic uncertainties and personal economic loss increased sharply during this time [38]. Thus, much of the declining SWB can be expected to be rooted in the aftermath of growing infection rates across the US. However, this effect may come with additional secondary health outcomes that may compound the relationship between cases and mood that worsen aggregate well-being over the course of the pandemic.

### COVID-19 and healthcare disparity

Although all cities in our analysis had an inverse relationship between COVID-19 infection and SWB, the effect was significantly more pronounced in cities with greater populations of color. This finding supports a disconcerting and now highly-documented observation that persons and communities of color are disproportionately affected by COVID-19 and its physical and secondary health effects [39–41]. This relationship is most pronounced in (Fig 1), which shows San Francisco, Nashville, and Seattle as cities with the highest SWB and lowest COVID-19 cases. The White population in those cities amounts to 40%, 65%, and 55%, respectively. By contrast, in Detroit, Miami and Chicago, where the White population comprises just 11%, 11%, and 28%, we observed the lowest SWB and highest COVID-19 infection rates. Importantly, higher COVID-19 rates in more diverse cities also correspond to national mortality rates by race, which is lowest among Whites (75.7 deaths per 100,000). Among communities of color, however, the mortality rate is (1) Black (123.7 deaths per 100,000); (2) Latino/a/x (86.7 deaths per 100,000); and (3) Native Americans (133 deaths per 100,000).

Broadly, this disparity may be explained by clinical factors, such as increased risk for Type-II diabetes and cardiovascular disease, which are predictors of severe COVID-19 infection [42]. However, communities of color are also prone to other psychosocial barriers to quality care including discrimination, risky occupations, and education, income, or basic access to healthcare and health insurance [43]. Thus, cities with greater minority populations are more likely to experience higher COVID-19 cases, which in our model is driven by race disparity rather than population size or density. This may, at least partially, explain lower SWB in racially diverse cities. However, we acknowledge other events and social drivers contributing to, and exacerbating, COVID-19 driven SWB declines.

### COVID-19 and diminished resilience

We performed an OLS regression to quantify the relationship between COVID-19 infections and SWB. In addition, we track longitudinal sentiment to examine how a city’s SWB changes over time [44], during the COVID-19 pandemic and whether changes in the relationship between SWB and COVID-19 cases can be induced by significant events. (Fig 2) tracks average VADER sentiment between January 22 through July 31, 2020. Overall, we observe a gradual decline in SWB, which supports findings from our OLS regression that the negative effects of the pandemic on SWB occur gradually over time, not sudden. However, through SWB tracking, we were able to capture events both related and unrelated to COVID-19 that were found to impact SWB: (1) Kobe Bryant’s death (January 26, 2020); (2) WHO declares COVID-19 a pandemic (March 11, 2020); and (3) George Floyd’s murder at the hands of a white police officer in Minneapolis, Minnesota (May 25, 2020). Separately, each event coincided with a stark and rapid decline in sentiment, which was most prominent after Mr. Floyd’s murder.

Intuitively, events can induce rapid declines in SWB that reflect in-the-moment changes in mood. However, acute events can also have a more lasting impact on SWB if SWB remains depressed after the event and the community’s SWB does not return to its previous baseline [45]. For example, many expressed sadness after Kobe Bryant’s death by sharing their grief through social media, after which SWB returned to previous levels within approximately 24 hours. However, after the WHO declared COVID-19 a pandemic, SWB took longer to stabilize, and did so at levels that were slightly lower than prior to the pandemic. And, after Mr. Floyd’s murder and collective moral injury [34], SWB, understandably, took even longer to stabilize, though at levels much lower than observed in early January. We note that collective effects on SWB after Mr. Floyd’s murder may have been particularly prominent in communities of color, thus compounding our findings on the relationship between COVID-19 cases, SWB, and community-level well-being. (Fig 3) shows the effect of Mr. Floyd’s murder on diminished subjective well-being. The slope of the linear fit remains similar before and after the event, but the y-intersect drops significantly. This may indicate that Mr. Floyd’s murder had a strong suppressing effect on overall well-being across all cities, from which recovery was only partial. Yet, the negative relation between mood and COVID-19 cases remained robust to different degrees, as evidenced by the changing scatter in (Fig 3). Some cities undergo larger drops in SWB than others because of this same event, again indicating a varying sensitivity possibly dependent on local factors such as a city’s demographics or news exposure.

### Limitations

This work is subject to limitations which we hope to address in future research. First, we stress that here our use of SWB as a proxy for mental health changes, following previous research [19], does not entail its use as a diagnostic indicator. Although considerable advances have been made in characterizing and diagnosing mental health changes from social media data [46] remains a fast-evolving challenge. This capability has recently been demonstrated in the literature by the use of supervised learning models [47] and natural language analytics [48], and may in future work be leveraged to perform stratified analysis of our cohort samples. We also acknowledge social media samples may skew younger, more educated, and less ethnically diverse than the general population [49]. However, significant pluralities of the US population [50] use some form of social media, with Twitter being used daily by over a fifth of all people in the United States [51]. We therefore contend that our sample is likely at least partially representative of the US population by virtue of its scope and scale. We also acknowledge that our regression models may be underspecified. There are other variables to consider that may explain variance in our model, including socioeconomic status (SES), poverty status, among others. We did not include these in our regression model given they fall beyond the scope of our research question, namely, to determine if demographic considerations predict COVID-19 cases and, by proxy, mood. We therefore strongly encourage future research to explore the link between COVID-19 and SES given the widely supported association between lower SES and increased COVID-19 cases. Finally, our study is observational, in nature, and it is thus difficult to generalize findings definitively and not suitable to draw causal inferences about the relation between mood and COVID-19 cases. However, the unprecedented scope and resolution of our analyses may contribute to a better understanding of the relationship between public mood and an important epidemic crisis.

## Concluding remarks on long-term mental health outlook amid a global pandemic

The pandemic and societal events may be associated with a diminished ability of communities to return to baseline SWB levels. This may suggest staggered pandemic fatigue that is having a lasting impact on SWB, and US mental health. Personal vulnerabilities and exposure to non-COVID-19 related stressors may also be at play in the diminished sentiment across specific communities. Similar links between social media, mood, and mood rebound, have particularly been studied during natural disasters, wherein sentiments expressed on social media decline in reaction to the event, but rebound shortly after the event has passed [46]. However, the COVID-19 pandemic presents evidence of diminished capacity to rebound, possibly since it is an ongoing and evolving crisis spanning multiple years. Future studies should continue this line of work by aggregating timelines even further to study continued mental health decline through late 2020 and beyond, particularly emphasizing race disparity and urban-related inequities beyond the COVID-19 pandemic.

## Supporting information

S1 Table Base model. We performed an OLS regression predicting average VADER scores on confirmed COVID-19 cases per 1,000 people in 10 US metropolitan cities. Our model was statistically significant, indicating confirmed COVID-19 cases (Log10 Signal) was associated with lower mood (β = -.017, 95% CI [-.03, -.006], p = 0.004, adjusted R^2^ = 0.34).
(DOCX)

S2 Table Regression model with all predictors. Base model with additional population independent variables. We then performed an OLS regression predicting VADER scores on confirmed COVID-19 cases per 1,000 people in 10 metropolitan cities. We also included the following IVs (1) city population (i.e., the total number of people residing in each metropolitan area), (2) population density (i.e., a measurement of population per unit area, or exceptionally unit volume), and (3) city demographics (i.e., percent white versus non-white). Confirmed COVID-19 cases (β = -.012, 95% CI [-.02, -.004], p = .006) and City Demographics (β = .063, 95% CI [.039, .087], p < .001, adjusted R^2^ = 0.81) were statistically significant.
(DOCX)
