# Supplementary material for: Declining well-being during the COVID-19 pandemic reveals US social inequities
Source: PLoS One. 2021 Jul 8;16(7):e0254114. doi: 10.1371/journal.pone.0254114 (PMC8266050; doi:10.1371/journal.pone.0254114)

S1 Table. *Base model.* We performed an OLS regression predicting average VADER scores on confirmed COVID-19 cases per 1,000 people in 10 US metropolitan cities. Our model was statistically significant, indicating confirmed COVID-19 cases (Log10 Signal) was associated with lower mood (β =-.017, 95% CI [-.03, -.006], p = 0.004, adjusted R^2^ = 0.34).


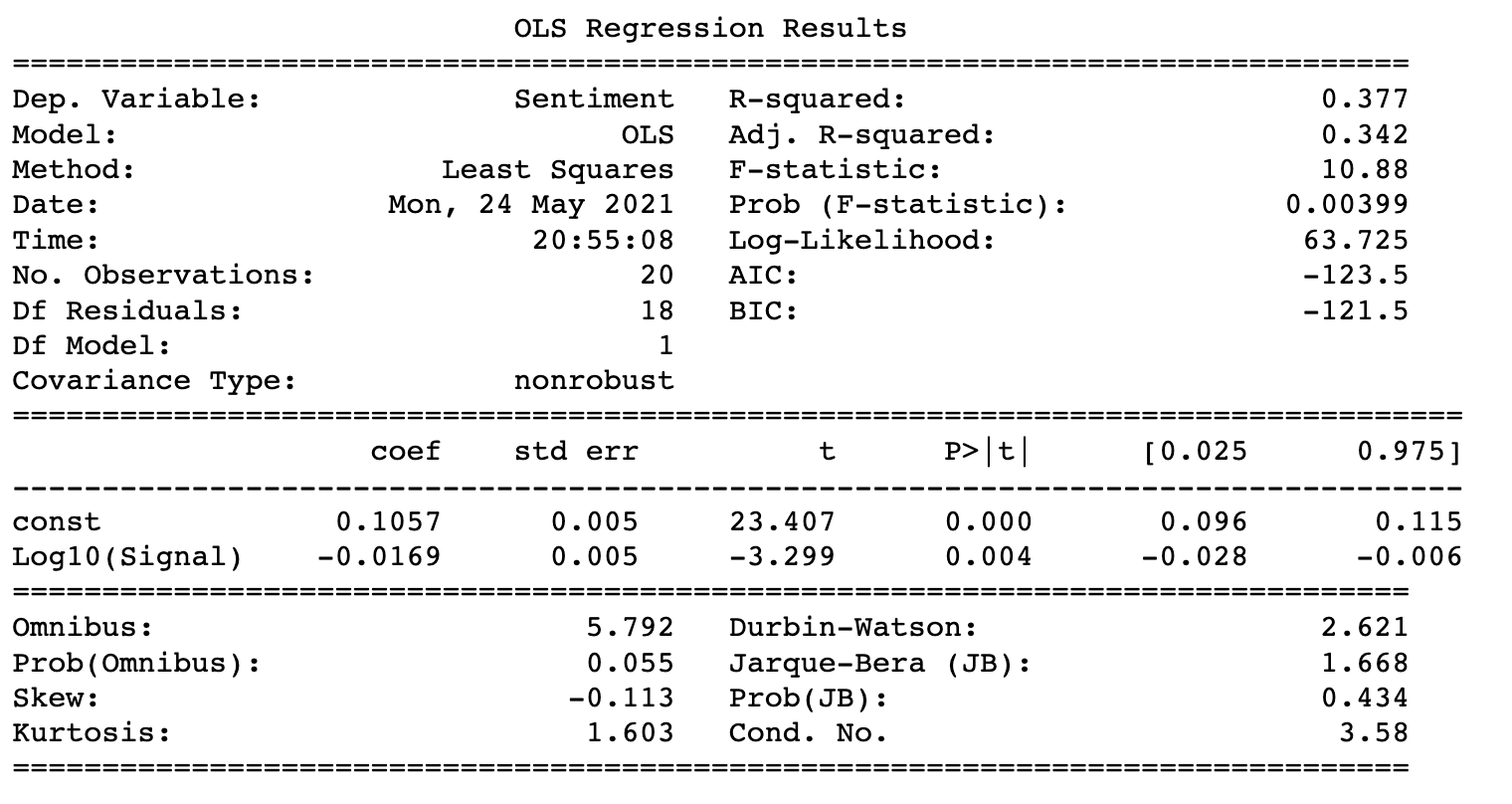

Supplement: S1 Table — We performed an OLS regression predicting average VADER scores on confirmed COVID-19 cases per 1,000 people in 10 US metropolitan cities. Our model was statistically significant, indicating confirmed COVID-19 cases (Log10 Signal) was associated with lower mood (β = -.017, 95% CI [-.03, -.006], p = 0.004, adjusted R2 = 0.34). (DOCX) [file pone.0254114.s001.docx]
