# Supplementary material for: Declining well-being during the COVID-19 pandemic reveals US social inequities
Source: PLoS One. 2021 Jul 8;16(7):e0254114. doi: 10.1371/journal.pone.0254114 (PMC8266050; doi:10.1371/journal.pone.0254114)

S2 Table. *Base model with additional population independent variables.* We then performed an OLS regression predicting VADER scores on confirmed COVID-19 cases per 1,000 people in 10 metropolitan cities. We also included the following IVs (1) city population (i.e., the total number of people residing in each metropolitan area), (2) population density (i.e., a measurement of population per unit area, or exceptionally unit volume), and (3) city demographics (i.e., percent white versus non-white). Confirmed COVID-19 cases (β =-.012, 95% CI [-.02, -.004], p = .006) and City Demographics (β =.063, 95% CI [.039, .087], p < .001, adjusted R^2^ = 0.81) were statistically significant.


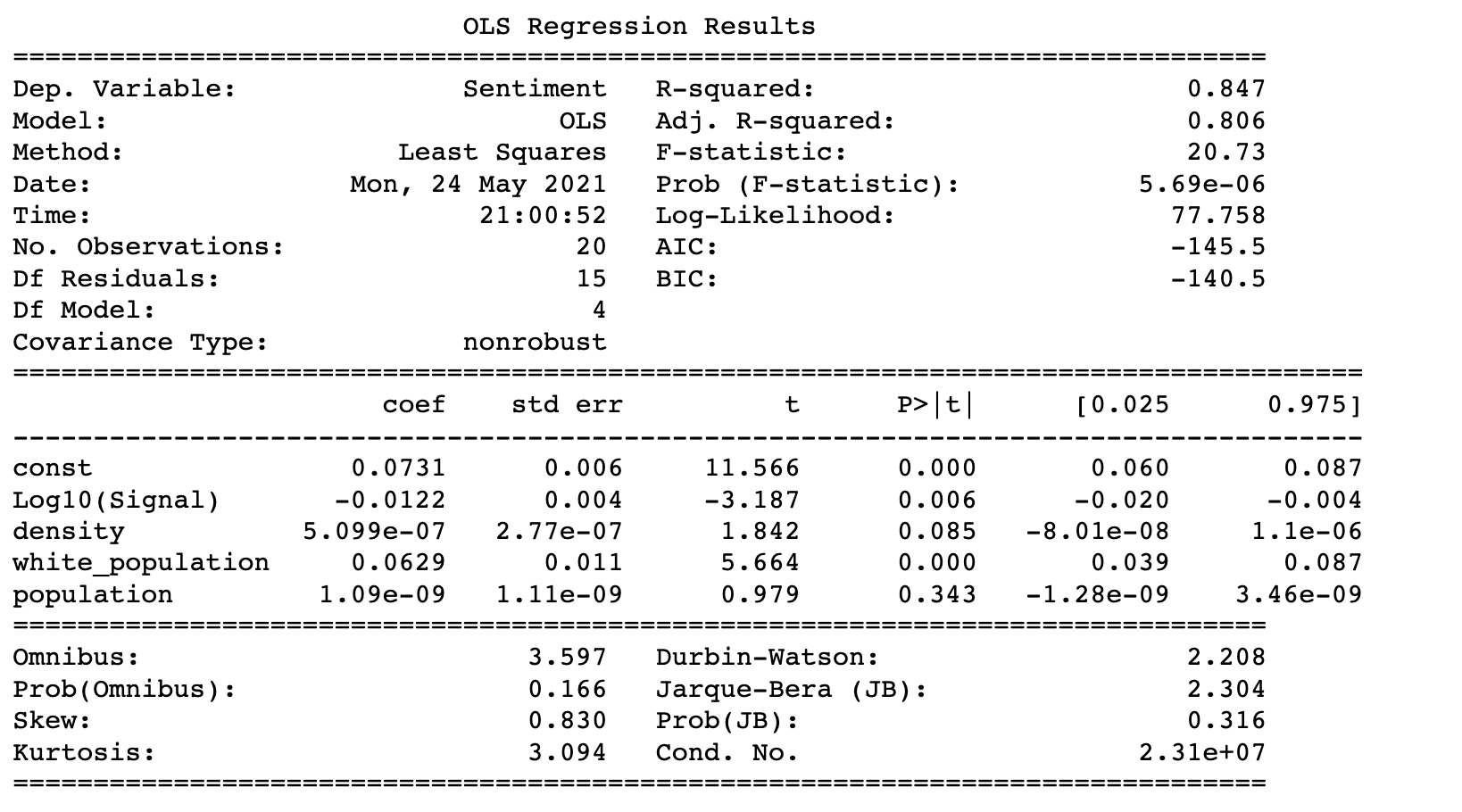

Supplement: S2 Table — Base model with additional population independent variables. We then performed an OLS regression predicting VADER scores on confirmed COVID-19 cases per 1,000 people in 10 metropolitan cities. We also included the following IVs (1) city population (i.e., the total number of people residing in each metropolitan area), (2) population density (i.e., a measurement of population per unit area, or exceptionally unit volume), and (3) city demographics (i.e., percent white versus non-white). Confirmed COVID-19 cases (β = -.012, 95% CI [-.02, -.004], p = .006) and City Demographics (β = .063, 95% CI [.039, .087], p < .001, adjusted R2 = 0.81) were statistically significant. (DOCX) [file pone.0254114.s002.docx]
